# Serial Position Learning in Honeybees

**DOI:** 10.1371/journal.pone.0004694

**Published:** 2009-03-04

**Authors:** Randolf Menzel

**Affiliations:** Institute Biology, Neurobiology, Freie Universität Berlin, Berlin, Germany; University of Queensland, Australia

## Abstract

Learning of stimulus sequences is considered as a characteristic feature of episodic memory since it contains not only a particular item but also the experience of preceding and following events. In sensorimotor tasks resembling navigational performance, the serial order of objects is intimately connected with spatial order. Mammals and birds develop episodic(-like) memory in serial spatio-temporal tasks, and the honeybee learns spatio-temporal order when navigating between the nest and a food source. Here I examine the structure of the bees’ memory for a combined spatio-temporal task. I ask whether discrimination and generalization are based solely on simple forms of stimulus-reward learning or whether they require sequential configurations. Animals were trained to fly either left or right in a continuous T-maze. The correct choice was signaled by the sequence of colors (blue, yellow) at four positions in the access arm. If only one of the possible 4 signals is shown (either blue or yellow), the rank order of position salience is 1, 2 and 3 (numbered from T-junction). No learning is found if the signal appears at position 4. If two signals are shown, differences at positions 1 and 2 are learned best, those at position 3 at a low level, and those at position 4 not at all. If three or more signals are shown these results are corroborated. This salience rank order again appeared in transfer tests, but additional configural phenomena emerged. Most of the results can be explained with a simple model based on the assumption that the four positions are equipped with different salience scores and that these add up independently. However, deviations from the model are interpreted by assuming stimulus configuration of sequential patterns. It is concluded that, under the conditions chosen, bees rely most strongly on memories developed during simple forms of associative reward learning, but memories of configural serial patterns contribute, too.

## Introduction

Learning of stimulus sequences requires memory of the temporal order of occurrences. Under natural conditions temporal sequence is often combined with spatial sequence, e.g. in navigational tasks. Position in space of objects is defined both by the temporal sequence of experience for the navigating animal and by its relation to surrounding cues, giving the position of each item a unique spatial character. Memories developed for sequential spatial positions of items may, therefore, be embedded in a large-scale relational spatial memory (a mental map). The memory formed under these conditions has been recognized as episodic or episodic-like resembling key features of memories that allow humans to mentally experience a previous occasion in space and time [1], [2]. One characteristic feature of episodic-like memory is the configuration of serial patterns into unique episodes [3]. It is not too far fetched to ask whether an insect like the honeybee is able to create episodic-like memory because bees are known to navigate with reference to a map-like spatial memory [4], perform configural forms of compound learning (such as positive and negative patterning in olfactory conditioning, [5]), master serial conditional discrimination like matching-to-sample and non matching-to-sample tasks [6], extract rules from multiple training sets (e.g. symmetrical vs asymmetrical patterns, [7] sequences of turn is mazes with multiple choice points, [8]), and organize their foraging activities according to multiple circadian time windows according to occasion setting conditions (review: [9], [10]). However, none of these experiments allowed rejecting more simple explanations, e.g the familiarity of signals, the recency of experience, differences of the strengths of memory traces and other characteristics of associative learning. In such a scenario one would expect the learning of the temporal-spatial sequences to be defined predominantly with respect to the evaluating conditions for the choice, the reward following the correct choice, and the fact that positions closer to the evaluating signal may have a greater impact on memory.

The role of order in a purely temporal sequences has been studied intensively after Ebbinghaus’ground braking discovery of the primacy and recency effect [11], [12]. Many examples are known meanwhile in which the last and the first items are better remembered than the middle items (bow-shaped memory function [13], [14]. According to the kind of errors made in recognizing the serial order of items (e.g. words, letters, numbers), several models were developed after Lashley's [15] account of creating a theoretical concept of serial order learning [16]. Such concepts range from assuming rather simple associative phenomena to specific coding of sequences of items, and it is generally agreed that a dominance of the recency effect does not require the assumption of a memory for the whole sequence.

Here we ask whether an insect, the honeybee, learns sequences of two colors in a context that attempts to simulate a navigational task. The bees fly in a T-maze which due to its narrow channels stimulates their neural distance measuring device (odometer) so strongly that they appear to experience multiples of the actual flight length [17]. They learn to turn right or left at the T-intersection according to the sequential color pattern they have experience during their flight in the access arm. In such a task temporal and spatial components of serial order are tightly connected and are associated with the outcome (reward) after a decision has been made based on serial discrimination. From a learning-theoretical point of view the task examined here belongs to those in which the animal has to discriminate patterns of compound stimuli (color, position, sequence) to form conditional discriminations (e.g. serial feature positive or negative tasks, [18]). If bees were to solve the task by some episodic-like or configural color sequence memory we would expect rather equal salience of the four sequential color positions and unique patterning effects reflecting memory of the whole or at least part of the sequential pattern. If, however, simple associative phenomena dominate their choice behavior one would expect a deviation from the bow-shaped function of the serial stimulus salience and a salience rank order that reflects the distance from the evaluating conditions (reward). It is known that sensory memory for visual and olfactory stimuli in reward learning in bees allows for an interval between the cue and the reward of several seconds [19], [20]. Since the four serial color signals in our experiments are experienced within a few seconds prior to the reward, both a configuration into a unique sequential pattern and a dominance of simple associative phenomena are possible. There is a rich literature on bees learning to associate particular signals to motor routines like moving right or left [21], [22]. In some of these experiments bees were also exposed to sequential signal/turn relations, and it was found that they learn multiple associations under particular training conditions [8], [23]. The sequences of signals tested in matching to sample (or matching to non-sample) paradigms [6], [20] need to be experienced within up to 5 sec before the match. None of these experiments have yet addressed the question of how bees evaluate positions of sequences of visual signals that are experienced within short intervals as guiding signals for alternative turns.

I find that bees learn to discriminate a series of two colors in four positions. Stimulus salience follows a rank order according to the distance of stimulus position from the choice point with highest salience to the closest position indicative of a recency effect. Discrimination is predicted to a large extent by positional salience, but divergence from this rule and data from the transfer tests also indicate configural phenomena.

## Methods

**Figure 1:**
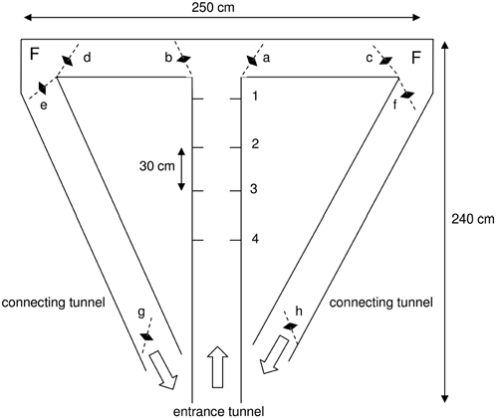
Bees approached the continuous T-maze from the hive at a distance of 35 m, entered it via the entrance tunnel, and proceeded through the entrance tunnel toward the T-junction after passing the 4 positions (1–4) of the color signals (either blue, B or yellow, Y). During training revolving doors (a–h) first guided the bees to enter the arm which provided food reward (F) at the end of the respective side arm, and, after sucking to completion, back out of the maze via the respective connecting tunnel. The bottom and walls of the tunnels were covered with a random black-and-white pattern, the structures of which appeared at a visual angle of approximately 10° when the bee flew in the middle of the tunnel. The top of the tunnel was made of UV-transmitting plexiglass. The color signals were arranged inside the entrance tunnel in such a way that the bees had to fly between two identical 10 cm color stripes.

Honeybees (*Apis mellifera carnica*) were trained from the hive to a T-maze (at a distance of 35 meters) during two summer periods. The T-maze was located between trees which allowed the bees a view of the canopy and the sky. The T-maze consisted of a 2.50 m long entrance tunnel (30×30 cm), two 1.10 m long tunnels to the right and left of the T intersection, and two 2.60 m long connecting tunnels that led the bees back to the entrance area after they had been feeding (continuous T-maze, Fig. 1). The bottom and the walls were covered with a random black-and-white pattern the structures of which appeared at a visual angle of approximately 10° when the bee flew in the middle of the tunnel. The top of the tunnel was made of UV-transmitting plexiglass. The color signals (either blue, B or yellow, Y) were arranged inside the entrance tunnel in such a way that the bees had to fly between two identical 10 cm color stripes (called here: signals). These color signals appeared at 4 different positions numbered 1, 2, 3 and 4 and were placed at distances of 30 cm. No. 1 was closest to the T-intersection (7 cm away from the T intersection). Bees were trained to fly in such a continuous T-maze. They entered it via the central tunnel (entrance tunnel), turned to the right or to the left at the T intersection, depending on the pattern of color signals at positions 1, 2, 3 and 4, and were rewarded at one of the two feeders (F). After feeding to completion, they flew out through one of the connecting tunnels. During the training session the flight path of the bee was guided by 8 revolving doors (a–h, Fig. 1).

A group of 5–9 bees shuttled regularly between the hive and the T-maze. They recruited newcomers, which became the experimental bees of the day. Two to four experimental bees were trained and tested only on the day of training. The group of recruiting bees was caged during the training and testing of the experimental bees. Sucrose concentration during training of the experimental bees was adjusted such that no further bees were recruited. The training of the experimental bees consisted of two phases: an initial phase lasting 60–90 minutes in which the bees learned to use only the entrance tunnel to access reward and to fly fluidly through the tunnel. No tests were performed during the initial training phase. In a second phase lasting 3–5 hours bees continued learning the particular arrangement of color signals at the four positions 1–4 which they had already experienced in the first training phase. Tests began when bees reached asymptotic performance after about 2 hours of training in the second phase. All bees mastered the task, and all bees trained were included in the tests. The flight time in the entrance tunnel was 3.2+/−1.8 s. Each experimental bee was trained to only one signal pattern, and all tests were performed on the day of training. In average each bee arrived 9 times during the initial training phase and 18 times during the second training/test phase.

Two kinds of tests were performed, within-training tests and between-training tests. In the first case food was available at the correct position, but both doors at the choice point were open. If the bee chose the correct arm of the maze, it was rewarded, and the choice was recorded as correct; if it chose the wrong arm it did not receive a reward, flew out through the connecting tunnel by closing the respective doors, and the choice was recorded as wrong. When it entered the entrance tunnel again it was guided to the feeder by closing the door into the wrong tunnel at the T intersection (no choice record). Thus any one bee made only one decision during a within-training test. During the between-training tests all doors were open, no feeder was available and the two feeder areas were covered with new paper of the same black-and-white random pattern. Bees were allowed to fly through the tunnel in any direction, but they usually flew into the tunnel via the entrance tunnel and exited via the respective connecting tunnel. Decisions were counted when a bee approached the T intersection via the entrance tunnel and flew at least half the length into one of the two arms of the T-maze. Each experimental bee made 3–5 choice flights during the between-training tests which lasted for 10 minutes. No difference was found between the results of the within-training and the between-training tests. Therefore, the data were pooled. Tests for the same signal patterns were repeated during the 3–5 hours of the test phase, and the different test patterns (including the transfer tests) followed each other in a pseudorandom fashion. Training for both patterns was continued during the test phase, and test patterns were always different from the last training pattern.

The serial position tasks involved two colors (B,Y) at four positions (1, 2, 3, and 4). Training patterns differed with respect to numbers and positions. The bee learned two patterns in sequential approach flights, one that was associated with a right turn in the T maze, and one associated with a left turn in the T maze. The notation of the training pattern will show the respective color (B, Y) at the respective position and the training side. Ø represents a position without a color signal. For example, [Ø B B Ø]r vs. [Ø Y Y Ø]l means bees learned to fly into the right arm when a blue signal appeared at positions 2 and 3 and no signal appeared at positions 1 and 4, and during the same training session the same bees learned to fly into the left arm when yellow signals appeared at positions 2 and 3 and none at positions 1 and 4. Table 1 and 2 summarize all experimental conditions, and tables S1 and S2 in the supplementary material give the choice data for all experiments.

**Table 1:** Summary of all discrimination experiments listing the training conditions, the patterns trained (for notation see Methods), the consecutive number of the experiment, the number n of choices, and the respective figure.

Column	Experiment	Training patterns	No. of experiment	Total number of choices	Figure
1	Differences in number and position	X Ø Ø Ø vs. X X Ø Ø	47a, 46	146	Fig. 2
2	“	X Ø Ø Ø vs. X X X Ø	48, 28	211	Fig. 2
3	“	X Ø Ø Ø vs. X X X X	47, 49	144	Fig. 2
4	“	Ø X Ø Ø vs. X X X X	2, 6	258	Fig. 2
5	One signal	B Ø Ø Ø vs. Y Ø Ø Ø	15	55	Fig. 3
6	“	Ø B Ø Ø vs. Ø Y Ø Ø	16, 44	154	Fig. 3
7	“	Ø Ø B Ø vs. Ø Ø Y Ø	17	30	Fig. 3
8	“	Ø Ø Ø B vs. Ø Ø Ø Y	18, 45	127	Fig. 3
	Two signals				
9	DS at different positions	12 different positions	25, 26, 27b, 30, 32, 33, 34, 50	424	Fig. 4A
10	SD at different positions	12 different positions	24, 23, 31, 20, 19a, 35, 51	405	Fig. 4B
11	DD at different positions	12 different positions	27a, 21, 22, 29, 19b, 52, 42	380	Fig. 4C
12	Three signals	D D D	36, 38	103	Fig. 5A
13	“	D D S	40, 9, 13	143	Fig. 5A
14	“	D S D	36a, 11	132	Fig. 5A
15	“	D S S	41, 10	105	Fig. 5A
16	“	S D D	14, 33a	119	Fig. 5B
17	“	S D S	37, 42a	120	Fig. 5B
18	“	S S D	12, 39	181	Fig. 5B

The consecutive experiment number indicates the sequence of experiments, assigning a new number to experiments performed on a different day. A number combined with a letter indicates that an additional experiment was performed on the same day. The first column (column Nr.) helps to coordinate this table with the table S1 and S2 in the supplementary material giving the choice values for all tests.

**Table 2:** Summary of all transfer experiments listing he training conditions, the patterns trained (for notation see Methods), the transfer patterns, the consecutive number of the experiment, the number n of choices, and the respective figure.

Column	Experiment	Training patterns	Transfer tests	No. of experiment	Total number of choices	Figure
1	Color transfers	Ø B Ø Ø vs. B B B B	Ø Y Ø Ø vs. Y Y Y Y	2	32	Fig. 6A
2	“	Ø Y Ø Ø vs. Y Y Y Y	Ø B Ø Ø vs. B B B B	6	19	Fig. 6B
3	“	Y B Ø Ø vs. B Y Ø Ø	B B Ø Ø vs. Y Y Ø Ø	21	56	Fig. 6C
4	“	B Y B Ø vs. Y B B Ø	B B B Ø vs. Y Y B Ø	9	70	Fig. 6D
5	Color and position transfer	Ø B Ø Ø vs. B B B B	Ø G G Ø G Ø Ø Ø	6	38	Fig. 7
6	Position transfer	B Ø Ø Ø vs. Y Ø Ø Ø	Four different patterns	15	46	Fig. 8A
7	“	Ø Y B Ø vs. Ø B Y Ø	Four different patterns	22	37	Fig. 8B
8	“	B B Ø Ø vs. Y B Ø Ø	Four different patterns	20	42	Fig. 8C
9	“	Y Y Ø Ø vs. B Y Ø Ø	Four different patterns	26	77	Fig. 8D
10	“	Ø B Y Ø vs. Ø Y Y Ø	Four different patterns	30	126	Fig. 8E
11	“	Ø B B Ø vs. Ø B Y Ø	Four different patterns	31	110	Fig. 8F
12	“	B Ø B Ø Y Ø B Ø	Two different patterns	33	48	Fig. 8G
13	Position transfer	B Ø Ø B vs. Y Ø ØB	Two different patterns	34	17	Fig. 8H
14	“	B Y B Ø vs. Y B B Ø	Two different patterns	9	34	Fig. 8I
15	“	B Y B Ø vs. Y B Y Ø	Two different patterns	36	55	Fig. 8J
16	Position and number transfer	Ø X Ø Ø vs. X X X X	Seven different patterns	2, 6	261	Fig. 9

The consecutive experiment number indicates the sequence of experiments, assigning a new number to experiments performed on a different day. A number combined with a letter indicates that an additional experiment was performed on the same day. The first column (column Nr.) helps to coordinate this table with the table S1 and S2 in the supplementary material giving the choice values for all tests.

Experimental design and statistics: Each test for a given pattern resulted in about 3 to 6 decisions made by each experimental bee, leading to up to 20 decisions per test. Tests for the same pattern were repeated several times for the same group of experimental bees on the same day, and many experiments were repeated with different experimental bees (see tables S1 and S2 in the supplementary material). The permutations of the three variables (color: B, Y; number of signals: 1–4; positions: 1–4) shall be presented in a systematic fashion but were carried out during two summer periods in an unsystematic way.

Statistic: To test pattern discrimination within a single experiment we used Fisher's exact tests. To analyze differences in performances across different experiments we used the G-test. We also used a paired t-test to compare the flight times for correct and incorrect choices, and Pearson correlation and linear regression to analyze the predictions of a model [24].

## Results

**Figure 2:**
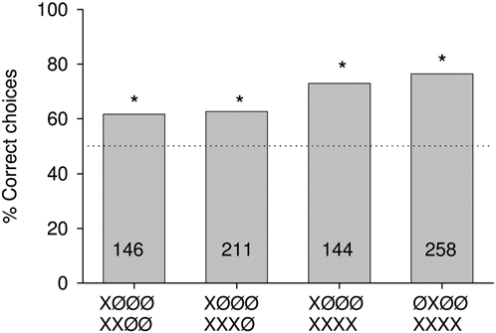
Percentage of correct choices for tasks in which bees were trained to discriminate between patterns offering signals of the same color but differing in number and position. X indicates either a blue or a yellow signal, but it was always the same color in the two alternatives (see text). Asterisks indicate significant discrimination between the training patterns: Fisher's exact test, P _XØØØ vs. XXØØ_ = 0.005, P _XØØØ vs. XXXØ_ = 0.0004, P _XØØØ vs. XXXX_<0.0001, P _ØXØØ vs. XXXX_<0.0001. Numbers inside the bars indicate the number of choices analyzed.

**Figure 3 pone-0004694-g003:**
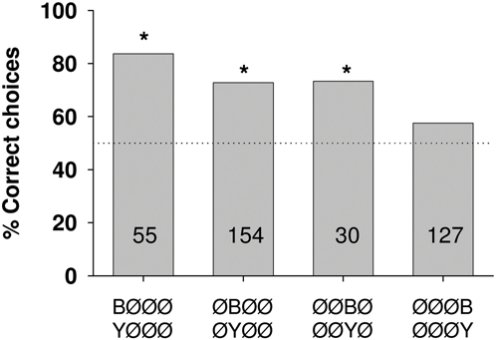
Percentage of correct choices for tasks in which bees were trained to discriminate between single signals of two different colors, B or Y. Asterisks indicate significant discrimination between the training patterns: Fisher's exact test; P _BØØØ vs. YØØØ_<0.0001, P _ØBØØ vs. ØYØØ_<0.0001, P _ØØBØ vs. ØØYØ_ = 0.004, P _ØØØB vs. ØØØY_ = 0.1. Numbers inside the bars indicate the number of choices analyzed.

**Figure 4 pone-0004694-g004:**
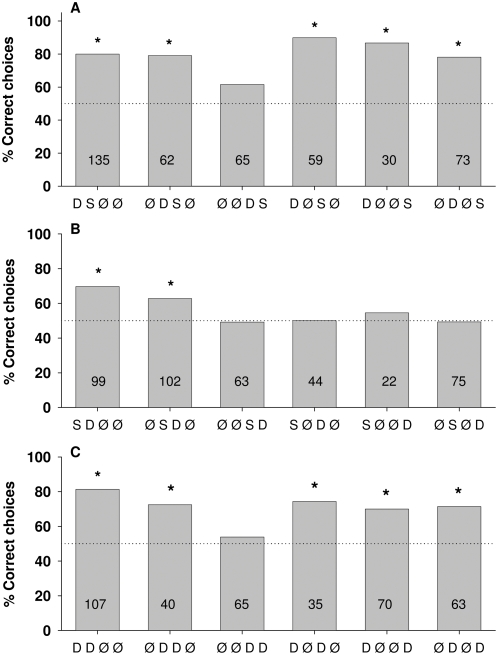
Percentage of correct choices for tasks in which bees were trained to discriminate between patterns offering two signals in the same position, this signal providing either the same (S) or different (D) colors. A, B and C show the six DS, SD and DD combinations respectively. Asterisks indicate significant discrimination between the training patterns: Fisher's exact test; A) P _DSØØ_<0.0001, P _ØDSØ_<0.0001, P _ØØDS_ = 0.07, P _DØSØ_<0.0001, P _DØØS_<0.0001, P _ØDØS_<0.0001. B) P _SDØØ_ = 0.0001, P _ØSDØ_ = 0.02, P _ØØSD_ = 0.07, P _SØDØ_ = 1, P _SØØD_ = 1, P _ØSØD_ = 1. C) P _DDØØ_<0.0001, P _ØDDØ_ = 0.01, P _ØØDD_ = 0.06, P _DØDØ_ = 0.007, P _DØØD_ = 0.002, P _ØDØD_ = 0.001. Numbers inside the bars indicate the number of choices analyzed.

**Figure 5:**
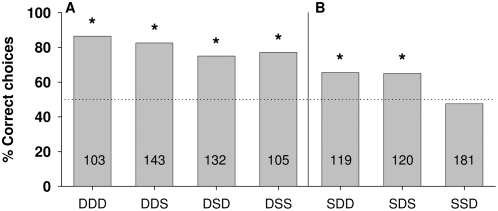
Percentage of correct choices for tasks in which bees were trained to discriminate between patterns offering three signals in the same position. These signals presented either the same (S) or different (D) colors. A Four combinations that presented a different signal in position 1. B Three combinations that presented a similar signal in position 1. Asterisks indicate significant discrimination between the training patterns: Fisher's exact test; A) P _DDD_<0.0001, P _DDS_<0.0001, P _DSD_<0.0001, P _DSS_<0.0001. B) P _SDD_ = 0.01, P _SDS_ = 0.01, P _SSD_ = 0.5‥ Numbers inside the bars indicate the number of choices analyzed.

The experimental design includes three variables, colors (B, Y), numbers (1–4), and positions (1–4). The role of these variables in guiding the bee in their choices at the T-intersection will be studied by systematically varying them independently and in combination. The measure of performance will be the probability of correct choices in discriminating the two patterns associated with the left and right turn (Chapter A: Discrimination tests, Fig. 2–5). In a first series of experiments (section A (1), Fig. 2) the color will be the same for both patterns but the numbers of signals and their positions will be different. Thus we first ask whether bees can use the numbers of signals to learn the turn in the T-maze. Then we shall vary the numbers of differently colored signals and ask whether the different positions provide the same or different salience for learning to orient in the T-maze. In addition, it will be interesting to search for any pattern effect possibly indicative for configural phenomena. I shall present the discrimination values first for one signal (section A (2), Fig. 3), then for two (section A (3), Fig. 4), and then for three signals (section A (4), Fig. 5).

**Figure 6:**
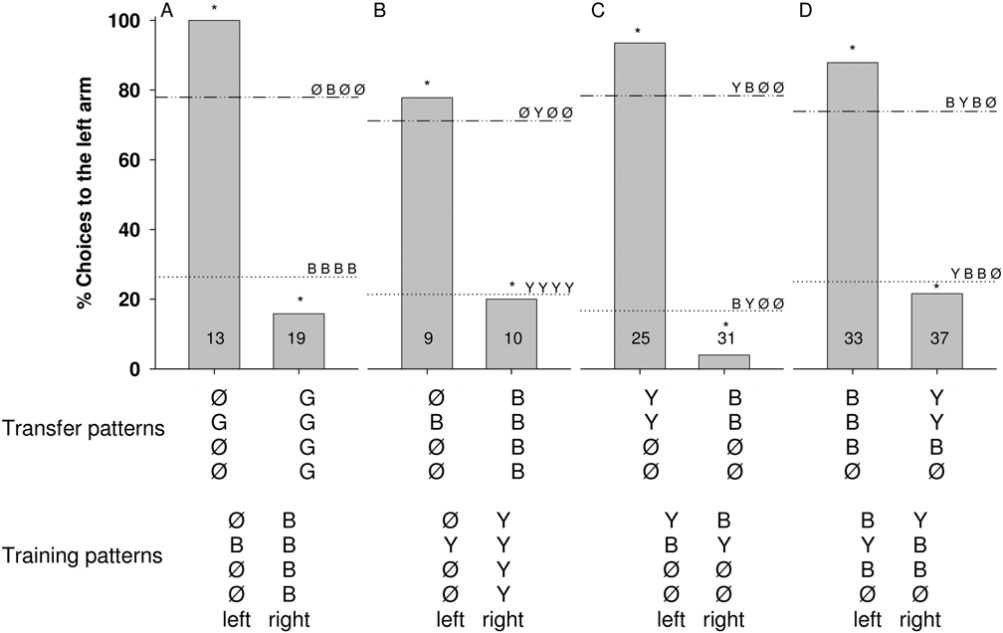
Percentage of choices toward the left arm of the maze for color transfer tests. Bees were trained to discriminate between patterns offering signals of the same color but differing in number and position (A, B), between patterns offering a DDØØ combination (C), or between patterns offering a DDSØ combination (D), and were then tested with transfer patterns which differed from the training patterns only in color. The trained patterns are given in the lower line and the transfer patterns in the line above. In the figure the choice values for the trained patterns are indicated by horizontal lines (…‥ or ‥_‥). The choice values for the transfer patterns are shown by columns. Asterisks indicate a significant transfer (i.e., the percentage of choices for a given transfer pattern significantly differ from only one of the training patterns and is similar to the other one): Fisher's exact test; A) P _ØGØØ vs ØBØØ_ = 0.07, P _ØGØØ vs BBBB_<0.0001, P _GGGG vs ØBØØ_<0.0001, P _GGGG vs BBBB_ = 0.4; B) P _ØBØØ vs ØYØØ_ = 1, P _ØBØØ vs YYYY_ = 0.004, P _BBBB vs ØYØØ_ = 0.002, P _BBBB vs YYYY_ = 1; C) P _BBØØ vs YBØØ_<0.0001, P _BBØØ vs BYØØ_ = 0.2, P _YYØØ vs YBØØ_ = 0.1, P _YYØØ vs BYØØ_<0.0001. D) P _BBBØ vs BYBØ_ = 0.1, P _BBBØ vs YBBØ_<0.0001, P _YYBØ vs YBBØ_ = 0.5, P _YYBØ vs BYBØ_<0.0001. Numbers inside the bars indicate the number of choices analyzed.

Configural phenomena of combinations of color, number and position may become apparent in generalization tests which ask whether the bee transfers a learned sequential pattern more strongly to one or another test pattern that was not experienced during training. A large number of such transfer tests will be presented in Chapter B (Transfer Tests, Fig. 6–9). Again the trained patterns will be systematically varied according to numbers and positions of these sequential patterns.

**Figure 7 pone-0004694-g007:**
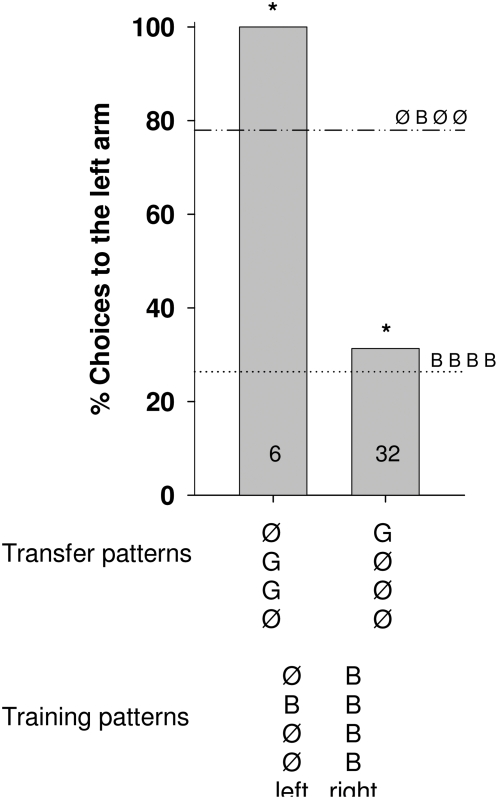
Percentage of choices toward the left arm of the maze for color, number and position transfer tests. The trained pattern is given in the lower line, and the transfer patterns in the line above. In the figure the choice values for the trained patterns are indicated by horizontal lines (…‥ or ‥_‥). The choice values for the transfer patterns are shown by columns. Asterisks indicate a significant transfer (i.e., the percentage of choices for a given transfer pattern differ significantly from only one of the training patterns and is similar to the other one): Fisher's exact test, P _ØGGØ vs ØBØØ_ = 0.34, P _ØGGØ vs BBBB_ = 0.0003, P _GØØØ vs BBBB_ = 0.6, P _GØØØ vs ØBØØ_<0.0001. Numbers inside the bars indicate the number of choices analyzed.

**Figure 8 pone-0004694-g008:**
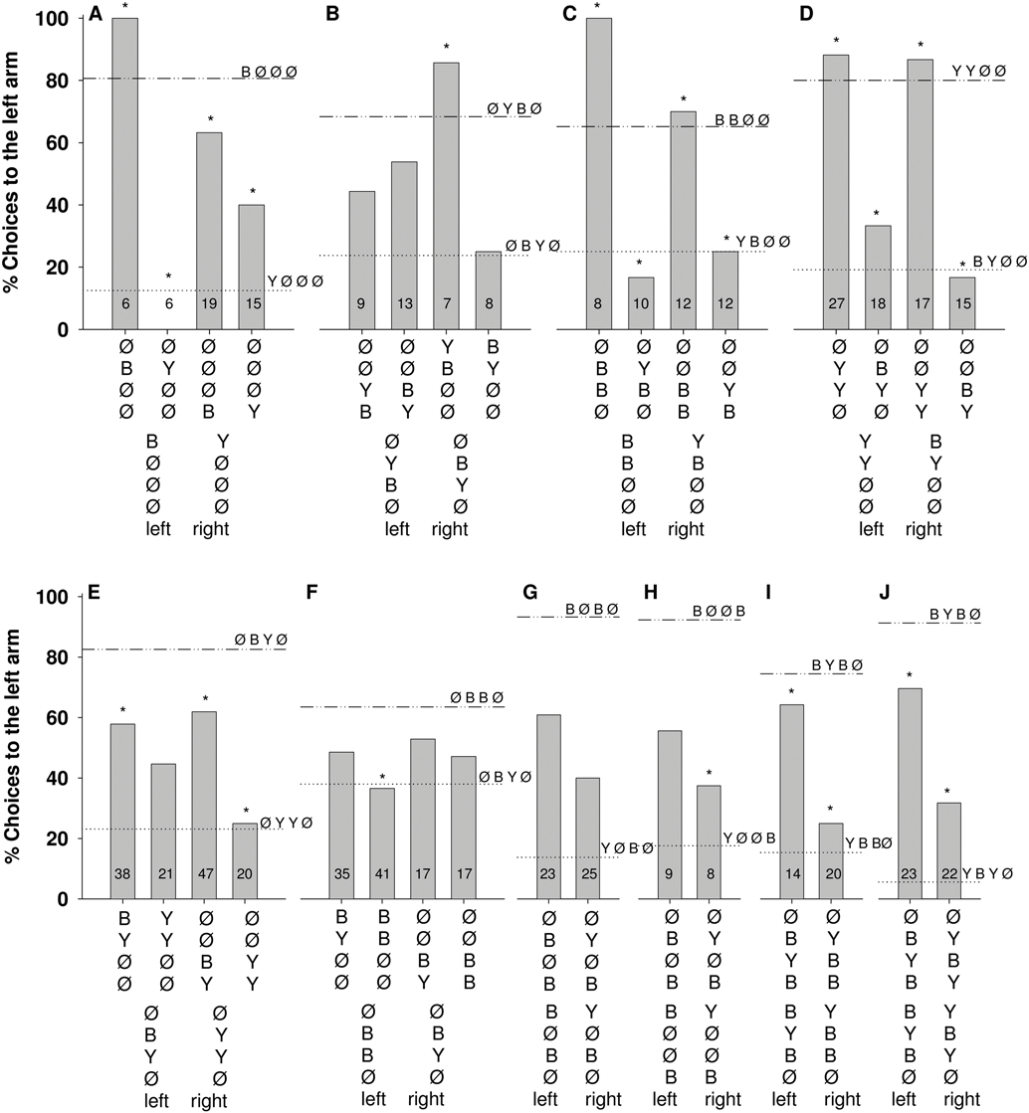
Percentage of choices toward the left arm of the maze for position transfer tests. A) Bees were trained to discriminate between patterns offering a single signal, either yellow or blue, in position 1 and tested with a pattern offering the single signal yellow or blue in positions 2 or 4. B, E, F) Bees were trained to discriminate between patterns offering a two signal yellow and blue in position 2, 3 and tested with a pattern offering the same signals in positions 3, 4 or 1, 2. C, D) Bees were trained to discriminate between patterns offering a two signal yellow and blue in position 1, 2 and tested with a pattern offering the same signals in positions 2, 3 or 3, 4. G) Bees were trained to discriminate between patterns offering a two signal yellow and blue in position 1, 3 and tested with a pattern offering the same signals in positions 2, 4. H) Bees were trained to discriminate between patterns offering a two signal yellow and blue in position 1, 4 and tested with a pattern offering the same signals in positions 2, 4. I, J) Bees were trained to discriminate between patterns offering a three signal yellow and blue in position 1, 2, 3 and tested with a pattern offering the same signals in positions 2, 3, 4. Numbers inside the bars indicate the number of choices analyzed. The trained pattern is given in the lower line, and the transfer patterns in the line above. In the figure the choice values for the trained patterns are indicated by horizontal lines (…‥ or ‥_‥). The choice values for the transfer patterns are shown by columns. A) P _ØBØØ vs BØØØ_ = 0.5, P _ØBØØ vs YØØØ_ = 0.0004, P _ØYØØ vs BØØØ_ = 0.001, P _ØYØØ vs YØØØ_ = 1, P _ØØØB vs BØØØ_ = 0.2, P _ØØØB vs YØØØ_ = 0.001, P _ØØØY vs BØØØ_ = 0.009, P _ØØØY vs YØØØ_ = 0.06. B) P _ØØYB vs ØYBØ_ = 0.4, P _ØØYB vs ØBYØ_ = 0.4, P _ØØBY vs ØYBØ_ = 0.5, P _ØØBY vs ØBYØ_ = 0.1, P _YBØØ vs ØYBØ_ = 0.6, P _YBØØ vs ØBYØ_ = 0.09, P _BYØØ vs ØBYØ_ = 1, P _BYØØ vs ØYBØ_ = 0.007.

**Figure 9:**
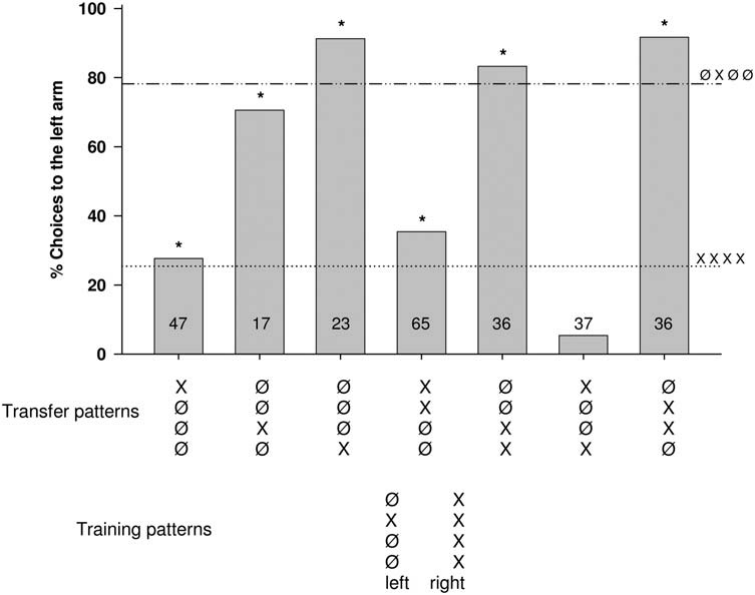
Percentage of choices toward the left arm of the maze for position and number transfer tests. Bees were trained to discriminate between patterns offering signals of the same color but differing in number and tested with pattern offering one or two signals of the same color in different positions. The trained pattern is given in the lower line, and the transfer patterns in the line above. In the figure the choice values for the trained patterns are indicated by horizontal lines (…‥ or ‥_‥). The choice values for the transfer patterns are shown by columns. Asterisks indicate a significant transfer (i.e., the percentage of choices for a given transfer pattern significantly differ from only one of the training patterns and is similar to the other one): Fisher's exact test; P _XØØØ vs ØXØØ_<0.0001, P _XØØØ vs XXXX_ = 0.8, P _ØØXØ vs ØXØØ_ = 0.5, P _ØØXØ vs XXXX_ = 0.0004, P _ØØØX vs ØXØØ_ = 0.2, P _ØØØX vs XXXX_<0.0001, P _XXØØ vs ØXØØ_<0.0001, P _XXØØ vs XXXX_ = 0.2, P _ØØXX vs ØXØØ_ = 0.6, P _ØØXX vs XXXX_<0.0001, P _XØØX vs ØXØØ_<0.0001, P _XØØX vs XXXX_ = 0.0006, P _ØXXØ vs ØXØØ_ = 0.09, P _ØXXØ vs XXXX_<0.0001. Numbers inside the bars indicate the number of choices analyzed.

The large number of possible combinations of the three parameters color, number and position can be reduced due to the fact that the two colors (B,Y) provide the same salience, and that the two directions of turns in the T-maze (right, left) are fully symmetrical.

### A. Discrimination tests

#### (1) Differences in numbers and positions

I first asked whether bees discriminate between two alternatives associated with right or left turns that differed in the numbers and positions of the signals, but not in their color. Fig. 2 gives the results for permutations in which one alternative was always one signal (either B or Y indicated by X) at either position 1 or 2, and the other alternative two, three or four signals. Different numbers are well discriminated even for a difference only by one signal in position 2, and discrimination improves if the alternatives differ in more than one signal. There is no difference between the choice values in experiments with two or three signals (each versus a single signal at position one) indicating that either position 3 contributes less to discrimination than position 1 and 2, or that the position effects are not cumulative. Taking the results of experiments with two or three signals together (each versus a single signal at position 1), and comparing them with those having four signals (again versus a signal at position one), a significant difference is found (P = 0.0208) indicating that the fourth position in the context of all four positions contributes to discrimination. In experiments with the single signal at either position 1 or 2 the same discrimination values indicate a similar salience of positions 1 and 2.

#### (2) One signal: differences in position and color

If a single signal differs in color for the two alternatives, bees learn the task particularly well when the signals are at position 1, equally well when at positions 2 and 3, but do not discriminate between the two alternatives when the signals are at position 4 (Fig. 3). Choice values are significantly higher for color difference at position 1 than those of the first and second bar in Fig. 2, indicating that positions 1 and 2 are most salient for discrimination. Thus large differences in numbers of signals (Fig. 2) have a lower salience than color differences if they appear at position 1 (Fig. 3). Color differences appearing at position 4 do not lead to significant discrimination (Fig. 3). Thus position 4 may not contribute to discrimination if animals are trained to use the color of a single signal.

#### (3) Two signals: differences in position and color

In the case of two signals at different positions, one can reduce the multitude of permutations to patterns in which the same position in the two alternatives presents either the same (S) or different (D) colors. Thus for positions 1 and 2 patterns of [B B Ø Ø] vs. [Y B Ø Ø], or [Y B Ø Ø] vs. [B B Ø Ø], or [Y Y Ø Ø] vs. [B Y Ø Ø], or [B Y Ø Ø] vs. [Y Y Ø Ø] belong to the same group of [D S Ø Ø] patterns. Since it was found that the two colors B and Y have the same salience, and no preference existed for one of the two sides, there was no need to test all possible permutations. Thus our notation for the three types of patterns DS, SD, DD at 6 permutations of 2 positions out of four is: DS at positions 1 and 2, 2 and 3, 3 and 4, 1 and 3, 1 and 4, 2 and 4 (Fig. 4A); SD at these same 6 positions (Fig. 4B), as well as DD at these same 6 positions (Fig. 4C).

Bees discriminate two signal patterns whenever differences appear in either position 1 or 2 or in both of these positions (Fig. 4). If the difference appears in position 3 then they may or may not discriminate the patterns (compare Fig. 4A, third column with Fig. 4 B, second and fourth column, and Fig. 4C, third column). The only case in which different signals in position 3 are discriminated (Fig. 4B second column) comes from experiments with a rather large number of decisions (n = 102). In all cases in which only the fourth position presented different signals, no significant discrimination between the two patterns is found (Fig. 4B last two columns). A possible indication of the rank order of salience in positions from 1 to 4 can also be seen when results from experiments are compared in which the signals are the same or different in one position, while other positions provided different signals. Consider the examples in which position 1 presents the difference and position 2 either a similar signal or no signal at all (Fig. 4A, C: [D S Ø Ø], [D Ø S Ø], [D Ø Ø S], [D Ø D Ø]. The highest scores are found for [D Ø S Ø] and [D Ø Ø S]. Although the respective discrimination scores are not significantly different, the discrimination scores follow [D Ø S Ø]>[D Ø Ø S]>[D Ø D Ø]) indicating that the similar signal at position 2 may reduce discrimination. This effect is also seen if position 1 presented a similar signal and position 2 presented a different one (Fig. 4B: [S D Ø Ø], as compared to Fig. 4A: [D S Ø Ø]: [D S Ø Ø]>[S D Ø Ø]). A similar tendency is seen for position 2 (Fig. 4 B: [Ø S D Ø], as compared to Fig. 4A: [Ø D S Ø]; [Ø D S Ø]>[Ø S D Ø], P = 0.04), and for position 3 if, for example, position 4 provides a different signal but position 3 a similar signal (Fig. 4B: [Ø Ø S D], as compared to Fig. 4A: [Ø Ø D S]; [Ø Ø D S]>[Ø Ø S D]). No such effect is seen for position 4 (Fig. 4C: [Ø Ø D D] as compared to Fig. 4A: [Ø Ø D S], P = 0.07).

The combined effect of differences and similarities in these two signal patterns on the rank order of positions 1–4 can be seen in a comparison of several other cases presented in Fig. 4. For example, comparing the discrimination scores in Fig. 4A with those in Fig. 4B indicates that the respective scores in Fig. 4A are, in most cases, significantly higher than those in Fig. 4B (Fisher's exact test: P_DSØØ vs SDØØ_ = 0.3; P_ØDSØ vs ØSDØ_ = 0.04; P_ØØDS vs ØØSD_ = 0.3; P_DØSØ vs SØDØ_<0.0001; P_DØØS vs SØØD_ = 0.01; P_ØDØS vs ØSØD_ = 0.0003). One would expect that different signals at both positions may add up and make it easier for the animal to discriminate the two alternatives. This is not the case, as a comparison between Fig. 4A and Fig. 4C shows. The scores given in Fig. 4C should be higher if the effect of differences were to add up, in fact, the corresponding patterns for D S versus D D are not significantly different (Fisher's exact test: P_DSØØ vs DDØØ_ = 0.8; P_ØDSØ vs ØDDØ_ = 0.5; P_ØØDS vs ØØDD_ = 0.6; P_DØSØ vs DØDØ_ =  0.08; P_DØØS vs DØØD_ = 0.1; P_ØDØS vs ØDØD_ = 0.4). This effect may indicate that the single signals at the 4 positions are not learned independently, but rather as a sequential pattern, which allows better discrimination if the two signal sequences differ when compared to similar sequential signals. This question will be further addressed with transfer tests (see below).

#### (4) Three signals: differences in position and color

Seven triple patterns are examined for the three positions 1, 2 and 3 ([D D D], [D S D], [D S S], [S D D], [S D S], [D D S] and [S S D]). Position 4 is excluded from these experiments because it was found earlier that signals at position 4 may not contribute at all or only marginally to discrimination. In each of the seven triple patterns, two experiments are performed which, because of the earlier findings showing that both colors and direction in the T-maze are interchangeable, are pooled in Fig. 5. The particular color/position assignments for these 14 experiments are given in the legend for Fig. 5. Again, higher discrimination scores are found for different signals at position 1. All scores in Fig. 5A are significantly higher than those in Fig. 5B, where the difference between those two groups of triple patterns is that those in Fig. 5A have patterns with different signals at position 1 and those in Fig. 5B have the same signals in position 1 (Fisher's exact test: P_D** vs S**_<0.0001). Differences only in position 2 are discriminated, but those in position 3 are not discriminated. The latter case is particularly interesting because the number of choices in these tests is rather high (n = 181).

A rank order of salience for positions 1 and 2 can be seen when comparing those triplets which differ only in position 1 or 2. Animals trained to a [DDD] situation show a significantly higher percentage of correct choices than the animals trained to a [SDD] situation (Fig 5A, G-test: P_DDD vs DSD_ = 0.0002). The same tendency is observed when position 2 provides the same signal and the other two positions the differences (Fig 5, G-test: P_DDD vs DSD_ = 0.027, after α level correction, differences should be taken as significant only if P<0.025). These results show that position 1 have the higher salience, followed by position 2. Position 3 does not contribute to discrimination in these triple patterns indicating that similar signals at positions 1 and/or 2 can overshadow the small contribution of position 3 as seen in the dual patterns (Fig. 4B).

### B. Transfer Tests

Hidden pattern effects may be uncovered by transfer experiments in which the color at a particular position (Fig. 6), or its position or the number of signals and their positions (Fig. 7–9) are changed in the test. Using such tests, we ask how well the animals are prepared to generalize from the learned to the transfer test stimulus conditions. Such transfer tests are always in between-training tests, because the animals are not rewarded under any of the test conditions. Since one cannot predict which choice (right or left) would be the correct choice, Figs. 6–9 plot the choice probability as a percentage of one direction (left).

#### (1) Color transfer tests

Color transfer tests are carried out after training to either equal or different numbers of signals in the two alternatives (Fig. 6). Bees transfer easily from blue to gray signals (Fig. 6A, Fig. 7; G indicates gray), or from yellow to blue signals (Fig. 6B–D). If the difference between the two signals during training lies in the positions 1 and 2 of the color, then bees decide according to the match of position 1 (Fig. 6C).

#### (2) Color, number and position transfer

The findings of the color transfer experiments are corroborated by results of transfer tests in which both color and position are changed (Fig. 7). Bees choose according to the match with the color of position 1, and appeared to ignore positions 2–4. If position 1 does not provide a color signal during the transfer test (Fig. 8A, C, D), but position 2 shows the color signal which the animal learned at position 1, the choice does not differ from the trained conditions, indicating that similar signals at position 1 in training and transfer patterns overshadow those in other positions. However, if position 1 does not provide any information in the transfer test the learned signal at position 1 is transferred to position 2.

#### (3) Position transfer

Systematically changing stimulus positions by keeping the number and the colors of the stimuli constant indicates several additional rules about the role of stimulus position. Well-discriminated patterns (Fig. 8B: [Ø Y B Ø] vs. [Ø B Y Ø], Fig. 8E: [Ø B Y Ø] vs. [Ø Y Y Ø]; Fig. 8G: [B Ø B Ø] vs. [Y Ø B Ø], Fig. 8H: [B Ø Ø B] vs. [Y Ø Ø B]) are less well discriminated if the respective patterns are moved backwards from the T-choice point, to higher position numbers (Fig. 8B to [Ø Ø Y B] and [Ø Ø B Y]; Fig. 8E to [Ø Ø B Y], Fig. 9G: [Ø B Ø B] and [Ø Y Ø B]; Fig. 8H to [Ø Y Ø B] and [Ø Y Ø B]. Position 3 is relevant for this position transfer because the training pattern [Ø B Y Ø ] is transferred to [Ø Ø B B ] and the training pattern [Ø Y Y Ø ] to [Ø Ø Y Y] (with a significant difference between the choices in the transfer patterns, P = .0001, Fisher exact test, see Fig. 8E).

#### (4) Position and number transfer

Combined transfer tests for positions and numbers of signals are run after three training pairs [Ø B Ø Ø] vs. [B B B B], [Ø Y Ø Ø] vs. [Y Y Y Y], and [B Ø Ø Ø] vs. [B B B Ø] (Fig. 9). The respective patterns for the two colors are pooled because no significant difference was found between the respective transfer tests, and these patterns are expressed in Fig. 9 as [ØX Ø Ø] vs [XXXX] for the training patterns, and [X Ø Ø Ø[ , [Ø Ø X Ø], [Ø Ø Ø X], [X X Ø Ø], [Ø Ø X X], [X Ø Ø X], [Ø X X Ø] for the transfer pattern). Position 1 again turned out to be the most important stimulus position (e.g. [X X Ø Ø] or [X Ø Ø X] or [X Ø Ø Ø] are chosen as the trained pattern [X X X X] and [Ø X X Ø] as the alternative trained pattern [Ø X Ø Ø]). This indicates that the animals do not interpret the signal in the trained pattern [Ø X Ø Ø] as being presented at position 1, but learned it as the signal for position 2. Patterns [X X Ø Ø] and [X Ø Ø X] are transferred to trained pattern [X X X X], and patterns [Ø Ø X Ø] and [Ø Ø Ø X] are transferred to [Ø X Ø Ø], indicating that either the animals always turn according to trained pattern [X X X X] if there is a signal at position 1 and according to trained pattern [Ø X Ø Ø] if there is no signal at position 1, or that the number of signals also play a role, irrespective of position. The significantly lower transfer to pattern [X X Ø Ø] than to pattern [X Ø Ø X] ( P = .006, Fisher's exact test) shows that bees learn to relate position 2 to the alternative trained pattern. Transfer of pattern [X Ø Ø X] to [X X X X] results in significantly higher choice values than the choice of the trained pattern [X X X X] (P = .03, Fisher's exact test), indicating that similar signals at position 2 in the trained patterns reduce the choice for the trained signals, and that position 4 contributes to the choice.

Stimulus conditions which are not learned ([Ø Ø B Y] vs [Ø Ø Y B]; [B B B Ø] vs [B Ø Ø Ø ]) do not lead to significant transfer (transfer pattern tested: [Ø Ø Y Y], [Ø Ø B B], [Y B Ø Ø ], [B Y Ø Ø ], data not shown). In another case of a non-significant training effect ([B B B B] vs [B Ø Ø Ø]) the transfer to [B Ø B Ø] (n = 53) is significantly different from those to [Ø B B Ø] (n = 31) and [Ø B B B] (n = 13) (P = .049, and P = .008 respectively, Fisher's exact test, data not shown) indicating that a transfer test can uncover a weak learning effect with respect to differences in signals at positions 2–4.

Fig. 8B, and Fig. 8F–J show cases in which either no significant transfer is found or the transfer is strongly reduced although the training patterns are well discriminated: Fig. 8B: [Ø Ø Y B] and [Ø Ø B Y] after training [Ø Y B Ø ] vs [Ø B Y Ø ]; Fig. 8F: [B Y Ø Ø], [Ø Ø B Y] and [Ø Ø B B] after training to [Ø B B Ø] vs (Ø B Y Ø]; Fig. 8G: [Ø B Ø B], [Ø Y Ø B] after training to [B Ø B Ø] vs [Ø V B Ø]; and Fig. 8H: [Ø B Ø B] after training to [B Ø Ø B] vs [Y Ø Ø B]. In all these cases the transfer patterns provide on the one side the same patterns as the trained pattern, but since it is shifted to a different position a mismatch results between the learned signals at the respective position. This indicates that the sequence in the pattern and the signal positions both play a role in transfer.

Taking all these transfer results together, we can derive the following rules: (1) Generalization to different colors is readily performed. In this case, decisions are made with reference to position and number of signals. (2) Signals at position 1 provide the highest salience, and a shift to position 2, 3 or 4 lead to a gradual reduction of salience. This gradient resembles the one found in the training experiments, which showed that position 1 has the highest salience, position 2 a somewhat lower one, position 3 a much lower one and position a very low salience. (3) Transfer is reduced or lost if position 1 provides the signal for discrimination during training, but is empty in the transfer patterns (Fig. 8G, H, I, J). (4) If discrimination learning is based on position 2, as in the case of the experiment shown in Fig. 9, the signals at position 2 override other criteria such as the number of signals. (5) Number matching can also guide choice behavior (Fig. 9). (6) Serial patterns appear to play a role, too, but the effect is small (See Fig. 8B, C, E, F).

### C. Model Calculation

#### (1) Modeling the positional salience scores (PSS)

The results from both the discrimination and the transfer experiments clearly show that serial position is the main parameter. The question I shall address next is how well the data can be understood by assuming a particular rank order of positional salience, and whether additional factors may guide the bee's decisions.

Position 1 provides the most salient signal and position 4 the least, if any. To address the question of whether position is the only relevant parameter I modeled the effect of positional salience on discrimination by assuming two relations between position and a positional salience score (PSS). The linear model relates positions 1, 2, 3, and 4 to the PSS of 1.0, .7, .4, and .1, and the stepwise model assumes an equal PSS of 1.0 for positions 1 and 2, .5 for position 3, and zero PSS for position 4. Since the animals learn the difference between the two signal patterns the model creates a cumulative PSS that takes into account the differences at the respective positions weighted with their respective salience scores. The PSS are calculated for the discrimination tests, transfer tests are not considered.

It is not obvious how the animals might have related the differences at each position. Therefore, two calculations of the cumulative differences in PSS are run. In calculation 1 (all signals were learned independently) it is assumed that animals learn each signal in both alternatives, weight them according to the PSS, and the cumulative differences in PSS result from the sum of the differences. No PSS is given to a position without a signal. For example (numbers in brackets are the respective PSS according to the linear model): [B (1.0) B (.7) B (.4) B (.1)] vs [B (1.0) Ø (0) Ø (0) Ø (0)] lead to differences in cumulative PSS: 0+.7+.4+.1 = 1.2. In calculation 2 (signals are learned with respect to each other) it is assumed that only those signals are learned that are different. The respective numbers for the same examples are (again according to the linear model): [B (1.0) B (.7) B (.4) B (.1)] vs [B (1.0) Ø (0) Ø (0) Ø (0)] lead to differences in cumulative PSS: 0+.7+.4+.1 = 1.2. The same two calculations are performed for the stepwise model. Since choice performance is expressed in % for each of the two alternatives and the model requires the calculation of differences between the choice performances, the % values are first linearized by calculating the corresponding probit values and then subtracting these probits. This gives a probit value for each pair of trained patterns.

**Figure 10:**
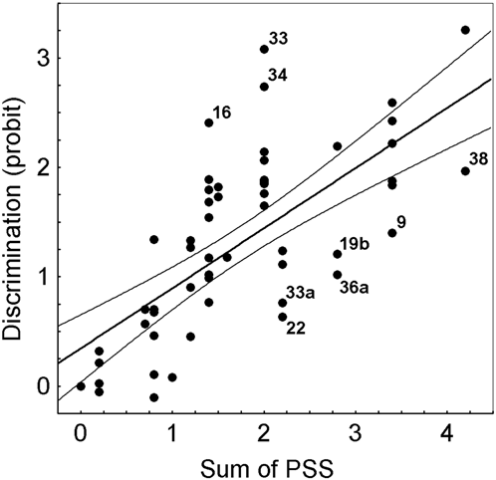
Linear regression between discrimination scores (ordinate) and the sum of position salience scores (PSS, abscissa) as calculated with the linear model applying calculation 1 (all signals are learned independently, see text). The discrimination scores (probit) result from converting the percentage of correct choices for each of the two alternative patterns into probit values and subtracting these probit values. The PSS for position 1 is set to 1.0, that of position 2 to .7, that of position 3 to .4, and that of position 4 to .1 (calculation 1, see text). Pearson correlation: P<0.0001, r^2^ = 0.49. The data points marked with the numbers of the respective experiments (see table 1) indicate outliers (selected by eye); numbers 16, 33 and 34 for experiments in which discrimination appears to be better than expected from the model calculation, numbers 9, 19b, 22, 33a, 36, 38 for experiments in which the discrimination scores appear to be lower than expected from the model calculation (see text). The thin lines show 95% confidence range.

The four models give the following results (Pearson correlation): linear model, calculation 1: P<0.0001, r^2^ = 0.49; linear model, calculation 2: P<0.0001, r^2^ = 0.48; step model, calculation 1: P<0.0001, r^2^ = 0.39; step model, calculation 2: P<0.0001, r^2^ = 0.41. The correlation coefficients for the four models are only marginally different. The best correlation is found for the linear model and calculation 1 (all signals are learned independently). This result is shown in Fig. 10. Although the model calculations show a correlation between the salience scores and discrimination, the rather low correlation coefficients do not allow distinguishing between the different assumptions behind these models.

Nine data points in Fig. 10 are marked as particularly clear deviations from close correlation. Three data points result from experiments in which performance appears to be better than expected from the model (exp. 16, 33, 34), and 6 data points in which performance is less than expected from the model (exp. 9, 19b, 22, 33a, 36a, 38). Better performance than expected is found if only one signal is provided (exp. 16: [Ø Ø B Ø] vs [Ø Ø Y Ø]) or if one of the two signals shows a difference in position 1 and the second signal is the same at either position 3 or 4 (exp. 33: [B Ø B Ø] vs [Y Ø B Ø]; exp. 34: [B Ø ØB] vs [Y Ø ØB]). Lower performance is found for serial signals which resemble components of a regular pattern in the two alternatives as, for example, reversed order (exp. 22: [Ø Y B Ø] vs [Ø B Y Ø]; exp. 36: [B B Y Ø] vs [Y B B Ø]; exp. 9: [Y B B Ø] vs [B Y B Ø]; exp. 38: [Y Y B Ø] vs [B B Y Ø]) or two equal signals in a series of three (exp. 33a: [Y Y B Ø] vs [Y B B Ø]). These results could indicate that bees may evaluate the 4 serial positions not only independently, but also to some degree as a pattern or a configural unit.

## Discussion

The task honeybees had to solve in these experiments was to fly either to the right or to the left in a T-maze to obtain food reward depending on two different patterns of up to 4 sequential blue or yellow signals. The sequential patterns were presented in a flight tunnel with black and white patterns on the floor and walls. The aim was to simulate a navigational task during a foraging episode, with the parameters involved in sequential landmark experience during approach flights more precisely controlled than it would be possible in a natural foraging range. Bees are known to interpret the length of their flights through a narrow tunnel as up to five times longer than their flights in the open due to their distance estimation via visual flow field [25], [26], [27], and they are also known to learn the sequence of landmarks on their foraging trips [9], [28], [8]. Therefore, it might well be that bees apply a form of observational or latent learning in such a simulated navigational task, and thus the sequential signals may be learned in spatial-temporal relation to each other, rather than in their temporal contiguity with respect to the reward. If the sequential signals would be learned in relation to each other with the potential to be grouped together to form unique configured experiences we expect rather equal salience of the various positions, and the appearance of phenomena indicative of stimulus configuration. If, however, associative learning dominates learning strategy the most recently experienced signal before reaching the reward might be weighted highest, and thus the salience of the sequentially experienced positions should be different. This is what I found. Position 1 closest to the intersection and the reward has the strongest impact on discrimination and generalization, position 2 provides close to equal salience as position 1, position 3 has a lower salience than position 2, and position 4 contributes only weakly or not at all to discrimination and generalization. Thus the temporal contiguity between the sequential signals and the reward appears to be the most important parameter. Configural phenomena are not absent but have a low impact (see below).

These results can be interpreted in several ways. I first consider the possibility that training in a narrow tunnel may not simulate a natural navigational situation. Several reasons may account for this effect. a) Similar context conditions: Although bees may experience longer flight distances in the tunnel the same location in the navigational space of the bee may not allow the bee to associate different serial positions of “landmarks” to different flight directions. This possibility is important because similar training conditions with bees flying over short distances in boxes or tunnels were used in the past to study questions related to navigation in bees (review: [29]). My results call into question whether such tests conditions allow generalization to the navigational context. This argument of caution may be particularly relevant for a recent study in which bees were trained to visual signals presented in a narrow tunnel, where these signals were denoted as “landmarks” [30]. b) Temporal order during learning: Bees as other animals [31] localize important items in time. They match their choices to the average of reward as experienced in multiple visits [32], [33], they monitor the gradient of reward over multiple visits [34], they activate time linked memories in diurnal rhythms [9], [35] and they learn to visit sequential feeding places at long distances during one foraging bout (unpublished observation). However, in all these cases temporal sequences appeared at much longer intervals (minutes to hours instead of seconds). Therefore, the temporal weighting of information may follow different rules of integration and memory formation. c) Temporal order during memory retrieval: The weight of recent experience is often higher in memory retrieval shortly after learning, whereas longer retrieval intervals favor may more balanced weights for sequential items [36] (see also below). Furthermore, the last learned item overshadows earlier learned items (bees: [37], [38]). These recency effects has been studied in the context of navigation for multiple phases of learning, and thus may not apply for the training conditions used here, but it will be necessary to ask in future experiments whether the recency effect seen here depends on the interval between learning and retention test. Taken together these arguments favor the conclusion that sequential signal learning in the T-maze does not mimic a navigational task.

It thus appears that the training conditions in the T-maze favor associative learning of multiple sequential stimuli experienced within the time span of sensory/working memory as tested in trace conditioning paradigms. Two parameters may be instrumental for the rank order of positional salience, limited time span and limited capacity of sensory/working memory. A multitude of associative learning phenomena favor temporal recency to the evaluating (reinforcing) stimulus (e.g. Pavlovian and instrumental conditioning, forgetting, recovery from extinction, delayed matching-to-sample, overshadowing, and others; review with respect to the bee: [39]). In bees the optimal CS-US interval is in the range of a few seconds both in instrumental color learning of freely flying animals and in odor conditioning of the proboscis extension response. Sensory memory can be extended to about 15 sec by an autoshaping procedure in which free-flying bees were trained in a dual forced-choice tests to expect delayed reward [40]. Since the bees experienced the 4 signals in the access arm of the T–maze within a few seconds (3.2+/−1.8 s) before arriving at the reward site I conclude that the time span for trace conditioning is not the limiting factor. It is thus more likely that limited capacity of the sensory store or cue competition between successively experienced stimuli is the critical factor.

Evidence for a limited capacity of the sensory store comes from matching-to-sample experiments which were carried out with two sequential visual stimuli under similar conditions as applied here [6], [20]. In such experiments it was found that bees learned to use 2 signals but not 3 for a later match, indicating that the storage capacity for item numbers may be very much limited. In experiments aiming to elucidate the question whether bees have some competence of counting (see below) the number of visual signals referred to reached 3 to 4. Thus the rank order of salience may reflect a sensory/working memory store limited to 3 items in bees.

One way of conceptualizing cue competition within sensory memory is to assume a form of overshadowing between stimuli. Overshadowing of stimuli equipped with different salience has been found in instrumentally trained [41] and in classical conditioned bees [42]. If sequential stimuli are equipped with different salience according to position, the first one experienced providing least salience, the last one experienced highest salience, the last ones will overshadow the first ones and association will be reduced accordingly. Another way of looking into cue competition would be to understand sensory memory as a shift register: whenever something new comes in a previously stored item has to leave (the first-in-first-out rule, see for example [14]). In any case it appears that the stimulus traces initiated sequentially during the approach flight may act on each other competitively rather than supporting each other as assumed, e.g. in the associative chaining or positional coding hypotheses [43].

Sequentially experienced signals have numerical attributes. Thus the question arises whether bees extract such attributes from the pattern of signals as tested here. The number of sequentially experienced landmarks was found to be a guiding factor in bee navigation [44], and it was concluded that bees judge distance flown not only on the basis of their visual odometer but also on the basis of the sequence and “number” of landmarks passed by. This capacity might reflect a basic form of precounting [45] which has been assigned to many animals including insects [46], [47], [48], [49], [50].

Recently the capacity of sequential numerosity has been demonstrated for bees flying in a tunnel, and passing by up to four signals at varying spatial separation [30]. This study showed that bees appear to learn up to three sequential signals, and since they transferred the trained “number” to novel signals it was concluded that they might perform some form of “exact counting”. Irrespective of the validity of this claim it is important in our context that the estimated upper limit of sequential signal numbers in a tunnel is three - a number that coincides with what I have found in the experiments presented here, and what is known form many studies in animals and humans (e.g. monkey: [51], human babies: [52]) . With respect to the question of whether my results are indicative of exact counting one might be skeptical because of the strong rank order of position salience. Although bees discriminate sequences of similar signals (Fig. 2) the effect is small, and the dominance of the signal in position 1 reduces the number effects in most other test conditions. The results of transfer tests after training [X X X X] versus [Ø X Ø Ø] (Fig. 9) could indicate that the numbers of signals are of importance, and that even position 4 may contribute to this effect. However, the transfer results could also be explained if one assumes that bees behave according to what they have learned about position 1 (with a signal in pattern [X X X X], no signal in pattern [Ø X Ø Ø]). Approximate counting, the discrimination of 1 vs 2, 2 vs 3, 2 vs 4 is supported by my data, but since the number effect is limited to 3 (or 4) it is not possible to test a characteristic property of approximate counting namely that it follows Weber's law (equal ratios are discriminated equally, [53]). Clues to numeric ability could be derived from the observation that sequential pattern effects are less strong in patterns with 3 signals as compared to those with two signals, indicating that configuration of sequential patterns may be limited to two signals. Thus the numerosity effect in associative learning as studied here may be limited to two, and is overshadowed by the strong position salience of position 1.

Sequences can be learned only if the animal keeps the temporal order in its sensory/working memory. It is well known from studies in humans and animals that sensory memory (also called primary, short-term or working memory with different emphasis on experimental paradigms) is limited in time and capacity. Serial memory tests have been used since James [54], Ebbinghaus [11] and Müller [55] to characterize the organization and temporal dynamics of this initial memory. Human subjects learning word lists and animals learning a series of visual features or landmarks show a serial position function (position meaning temporal position in most test conditions) with high retention scores at the beginning and the end of the series (primacy and recency effect, [14]. However, such a U-shaped retention function may not be the general case, since different stimuli (visual or auditory), different training-test intervals and different animal species may give rather divergent results. Under certain conditions the recency effect is favored, under other conditions the primacy effect, and sometimes both effects are detectable (U-shape function). For example Wright [56] [57] found that the recency effect dominates for short retention intervals ( 0–2 sec), and the primacy effect dominates for longer retention intervals (20 s, 30 s). Different animal species and different stimulus modalities influence the shape of the positional retention function (review [16]). The data reported here document the recency effect. This is in line with the observation that short retention test intervals favor the recency effect.

Next I want to ask whether there is any evidence for configural phenomena in the T-maze serial signal learning task. Since the signals are experienced well within the time span of working memory one might expect that these signals might be grouped together to form a unique compound. Although the serial salience effect is strong there is evidence that bees indeed learn the sequential patterns also as stimulus sequence patterns. (1) A comparison of the results in Fig. 4A and Fig. 4C shows that the discrimination scores for signals in positions 1 and 2 (Fig. 4C) do not add up and, in some cases, the scores are even lower than the respective ones for patterns with a difference only in position 1 (Fig. 4A). Discrimination is reduced if the two sequences have common features, whereas signals are better discriminated if they differ in pattern features. Thus signals at the positions 1, 2 and (to some extent) 3 are not only learned independently, but also as a sequential pattern indicating that these sequences are also learned as configured patterns. (2) Transfer experiments show that patterns are learned for their respective relative positions, and signals are not only weighted according to the positional salience rank order. For example, the transfer pattern [Ø X X Ø] is chosen more strongly than the trained pattern [X X Ø Ø ] (Fig. 8C), and the transfer pattern [X X Ø Ø] is less strongly chosen than the trained pattern [ØXX Ø] (Fig. 8E). (3) Although the model calculation on the basis of positional salience of the isolated signals captures most of the data quite well, I selected a few examples in which better performance or worse discrimination than expected was found (Fig. 10). Sequential patterns were better discriminated if only one signal provided the difference (exp. 16: [Ø Ø B Ø] vs [Ø Ø Y Ø]) possibly indicating a lack of interference from other signals. Better discrimination was also seen if one of the two signals showed a difference in position 1 and the second signal was the same at either position 3 or 4 (exp. 33: [B Ø B Ø] vs [Y Ø B Ø]; exp. 34: [B Ø ØB] vs [Y Ø ØB]). This effect may hint at a priming effect of the first signal encountered: the discriminative signal might receive additional value. Lower performance was found for serial signals which resembled components of a regular pattern in the two alternatives as, for example, reversed order (exp. 22: [Ø Y B Ø] vs [Ø B Y Ø]; exp. 36: [B B Y Ø] vs [Y B B Ø]; exp. 9: [Y B B Ø] vs [B Y B Ø]; exp. 38: [Y Y B Ø] vs [B B Y Ø]) or two equal signals in a series of three (exp. 33a: [Y Y B Ø] vs [Y B B Ø]). Taken together these results indicate that bees learn these sequential patterns to some extent as configural units in addition to their isolated functions. Training against the dominance of the positional rank order may allow isolating these configural components. Configuration of sequential patterns in mammals has been interpreted as indicating relational representations in time and space [58], [59]. The structure of such representations resemble key components of episodic-like memory, e.g. the configuration of 5 sequentially experienced odors as a unique episode. It will be a question for future experiments to test whether a comparable memory structure exists in an insect, the honeybee.

## Supporting Information

Table S1 Discrimination tests. The table gives the choice data of all experiments for discrimination tests. Rows (column a) are numbered as in Table 1 (test). Column b gives the number of the experiment as in Table 1, and the number of tests performed in the particular experiment. Column c shows the training pattern, first for the training to the left side of the T-maze, and then for the right site. Column d gives the number of animals trained and tested in the respective experiment. Column e gives the choices summed up for all tests first for the choice of the left arm and then the choice of the right arm of the T-maze. Column f gives the % of correct choices.
(0.07 MB DOC)

Table S2 Transfer tests. Rows and columns a, b, c and d are the same as in table S1. Column e gives the patterns presented during the transfer tests together with the choices for these patterns. As in table S1 the choices of the left arm are shown first and then the ones for the right arm of the T-maze. Column f gives the % of choices for the left arm.
(0.05 MB DOC)

## References

[1] Tulving E (1972). Episodic memory: From to brain.. Ann Rev Psychol.

[2] Clayton NS, Dickinson A (1998). Episodic-like memory during cache recovery by scrub jays.. Nature.

[3] Eichenbaum H, Cohen N (2001). From Conditioning to Conscious Recollection: Memory Systems of the Brain.

[4] Menzel R, Greggers U, Smith A, Berger S, Brandt R (2005). Honeybees navigate according to a map-like spatial memory.. Proceedings of the National Academy of Sciences of the USA.

[5] Deisig N, Lachnit H, Sandoz JC, Lober K, Giurfa M (2003). A modified version of the unique cue theory accounts for olfactory compound processing in honeybees.. Learn Mem.

[6] Giurfa M, Zhang SW, Jenett A, Menzel R, Srinivasan MV (2001). The concepts of ‘sameness’ and ‘difference’ in an insect.. Nature.

[7] Giurfa M, Zhang SW, Jenett A, Boenisch C, Wiley C (2001). The concepts of sameness and difference in an insect..

[8] Zhang SW, Mizutani A, Srinivasan MV (2000). Maze Navigation by Honeybees: Learning Path Regularity.. Learning & Memory.

[9] von Frisch K (1967). The dance language and orientation of bees.

[10] De Marco R, Menzel R, Menzel R (2008). Learning and Memory in Communication and Navigation in Insects.. Learning and Memory - A Comprehensive Reference: Vol. 1: Learning Theory and Behavior.

[11] Ebbinghaus H (1913). Memory: A Contribution to Experimental Psychology (translated from the 1885 German original by H.A. Ruger and C.E. Bussenius).

[12] Ebbinghaus M (1885). Über das Gedächtnis.

[13] Healy AF (1975). Coding of temporal-spatial patterns in short-term memory.. Journal of Verbal Learning and Verbal Behavior.

[14] Baddeley AD, Hitch G, Bower GH (1974). Working memory.. The psychology of learning and motivation: advances in research and theory.

[15] Lashley KS, Jeffress LA (1951). The Problem of Serial Order in Behavior.. Cerebral Mechanisms in Behavior.

[16] Healy AF, Bonk WJ (2008). Serial learning..

[17] Srinivasan MV, Zhang SW, Bidwell NJ (1997). Visually mediated odometry i in honeybees.. Journal of Experimental Biology.

[18] Holland PC, Bouton ME (1999). Hippocampus and context in classical conditioning.. Curr Op Neurobio.

[19] Menzel R (1999). Memory dynamics in the honeybee.. J Comp Physiol [A].

[20] Zhang S, Bock F, Si A, Tautz D, Srinivasan M (2005). Visual working memory in decision making by honey bees.. Proceeding of the National Academy of Sciences of the USA.

[21] Collett TS, Fry SN, Wehner R (1993). Sequence learning by honeybees.. J Comp Physiol [A].

[22] Collett TS, Rees JA (1997). View-based navigation in Hymenoptera: multiple strategies of landmark guidance in the approach to a feeder.. J Comp Physiol [A].

[23] Zhang SW, Bartsch K, Srinivasan MV (1996). Maze learning by honeybees.. Neurobiol Learn & Mem.

[24] Zar JH (1984). Biostatistical analysis.

[25] Esch HE, Zhang SW, Srinivasan MV, Tautz J (2001). Honeybee dances communicate distances measured by optic flow.. Nature.

[26] Srinivasan MV, Zhang SW, Altwein M, Tautz J (2000). Honeybee Navigation: Nature and Calibration of the “Odometer”.. Science.

[27] De Marco RJ, Menzel R (2005). Encoding spatial information in the waggle dance.. J Exp Biology.

[28] Chittka L, Geiger K, Kunze J (1995). The influences of landmarks on distance estimation of honey bees.. Anim Behav.

[29] Collett T, Paul Graham, Robert AHarris, Natalie Hempel-de-Ibarra (2006). Navigational Memories in Ants and Bees: Memory Retrieval when Selecting and Following Routes.. Advances in the Study of Behaviour Vol. 36.

[30] Dacke M, Srinivasan MV (2008). Evidence for counting in insects 1.. Anim Cogn early electronic publ.

[31] Gallistel CR (1990). The Organization of Learning.

[32] Greggers U, Menzel R (1993). Memory dynamics and foraging strategies of honeybees.. Behav Ecol Sociobiol.

[33] Fülöp A, Menzel R (2000). Risk-indifferent foraging behaviour in honeybees.. Animal Behaviour.

[34] Gil M, De Marco RJ, Menzel R (2007). Learning reward expectations in honeybees.

[35] Prabhu C, Cheng K (2008). One day is all it takes: Circadian modulation in the retrieval of color memories in honeybees.. Behav Ecol Sociobiol.

[36] Devenport LD, Devenport JA (1994). Time-dependent averaging of foraging information in leastchipmunks and golden-mantle ground squirrels.. Anim Behav.

[37] Cheng K, Wignall AE (2006). Honeybees (*Apis mellifera*) holding on to memories: response competition causes retroactive interference effects.. Animal Cognition.

[38] Menzel R (1969). Das Gedächtnis der Honigbiene für Spektralfarben. II.Umlernen und Mehrfachlernen.. Z vergl Physiol.

[39] Menzel R, Brembs B, Giurfa M, Kaas JH (2007). Cognition in Invertebrates.. Evolution of Nervous Systems, Vol. II: Evolution of Nervous Systems in Invertebrates.

[40] Grossmann K (1970). Erlernen von Farbreizen an der Futterquelle durch Honigbienen während des Anfluges und während des Saugens.. Z Tierpsychol.

[41] Couvillon PA, Klosterhalfen S, Bitterman ME (1983). Analysis of overshadowing in honeybees.. Journal of Comparative Psychology.

[42] Bitterman ME, Menzel R, Fietz A, Schäfer S (1983). Classical conditioning of proboscis extension in honeybees (Apis mellifera).. Journal of Comparative Psychology.

[43] Crowder CG (1968). Evidence for the chaining hypothesis of serial verbal learning.. J Exp Psychol.

[44] Chittka L, Geiger K (1995). Can honeybees count landmarks?. Anim Behav.

[45] Gallistel CR, Gelman R (2000). Non-verbal numerical cognition: From the reals to the integers.. Trends Cogn Sci.

[46] Shettleworth SJ (1998). Cognition, Evolution and Behavior.

[47] Brannon EM, Terrace HS, Bekoff M, Allen C, Burghardt 370GM (2008). The evolution and ontogeny of ordinal numerical ability.. The Cognitive Animal: Empirical and TheoreticalPerspectives on Animal Cognition.

[48] Boisvert MJ, Sherry DF (2006). Interval timing by an invertebrate, the bumble bee Bombus impatiens.. Curr Biol.

[49] Chen L, Zhang S, Srinivasan MV (2003). Global perception in small brains: Topological pattern recognition in honey bees.. PNAS.

[50] Skorupski P, Chittka L (2008). Animal cognition: An insect's sense of time?. Curr Biol.

[51] Hauser MD (2000). Homologies for numerical memory span?. 6. Trends in Cognitive Sciences.

[52] Starkey P, Spelke ES, Gelman R (1990). Numerical Abstraction by Human Infants 63.. Cogni.

[53] Meck WH, Church RM (1983). A Mode Control Model of Counting and Timing Processes 3.. Journal of Experimental Psychology-Animal Behavior Processes.

[54] James W (1890). The Principles of Psychology.

[55] Müller GE, Pilzecker A (1900). Experimentelle Beiträge zur Lehre vom Gedächtnis.. Z Psychol.

[56] Wright AA (1999). Visual list memory in capuchin monkeys (Cebus apella).. J Comp Psychol.

[57] Wright AA (2007). An experimental analysis of memory processing 2.. Journal of the Experimental Analysis of Behavior.

[58] Agster KM, Fortin NJE (2002). The hippocampus and disambiguation of overlapping sequences.. J Neurosci.

[59] Eichenbaum H (2002). The cognitive neuroscience of memory.

