# Supplementary material for: Serial Position Learning in Honeybees
Source: PLoS One. 2009 Mar 4;4(3):e4694. doi: 10.1371/journal.pone.0004694 (PMC2649506; doi:10.1371/journal.pone.0004694)
Supplement: Table S1 — Discrimination tests. The table gives the choice data of all experiments for discrimination tests. Rows (column a) are numbered as in Table 1 (test). Column b gives the number of the experiment as in Table 1, and the number of tests performed in the particular experiment. Column c shows the training pattern, first for the training to the left side of the T-maze, and then for the right site. Column d gives the number of animals trained and tested in the respective experiment. Column e gives the choices summed up for all tests first for the choice of the left arm and then the choice of the right arm of the T-maze. Column f gives the % of correct choices. (0.07 MB DOC) [file pone.0004694.s001.doc]

Table S1: Discrimination tests

| a | b | c | d | e | f |
| --- | --- | --- | --- | --- | --- |
| column in  Table 1 A | number of experiment:  number of tests | training patterns  (left vs right site) | number  of animals | sum of choices in all tests (left/right) | % of  correct choices |
| 1 | 46: 4 tests  47a: 4 tests | B000 vs BB00  Y000 vs YY00 | 3  2 | B000: 25/14  BB00:18/24  Y000:19/12  YY00:12/22 | 64%  57%  61%  65% |
| 2 | 28: 5 tests  48: 3 tests | BBB0 vs B000  Y000 vs YYY0 | 3  2 | BBB0: 45/38  B000: 18/30  Y000: 22/11  YYY0: 12/23 | 54%  62%  67%  66% |
| 3 | 47: 4 tests  49: 4 tests | B000 vs BBBB  Y000 vs YYYY | 2  2 | B000: 26/10  BBBB: 9/25  Y000: 25/10  YYYY:10/29 | 72%  74%  71%  74% |
| 4 | 2: 8 tests  6: 3 tests | 0B00 vs BBBB  0Y00 vs YYYY | 2  2 | 0B00: 56/9  BBBB: 24/57  0Y00: 19/5  YYYY: 6/22 | 86%  71%  79%  79% |
| 5 | 15 : 3 tests | B000 vs Y000 | 2 | B000: 25/6  Y000: 3/21 | 68%  87% |
| 6 | 16: 4 tests  44: 5 tests | 0B00 vs 0Y00  0Y00 vs 0B00 | 2  2 | 0B00: 26/7  0Y00: 4/14  0Y00: 33/18  0B00: 13/29 | 87%  86%  67%  69% |
| 7 | 17: 2 tests | 00B0 vs 00Y0 | 2 | 00B0: 18/5  00Y0: 6/13 | 78%  68% |
| 8 | 18; 2 tests  45: 4 tests | 000B vs 000Y  000B vs 000Y | 2  2 | 000B: 12/7  000Y: 6/9  000B: 25/20  000Y: 21/27 | 56%  40%  56%  56% |
| 9 | 25: 3 tests  26: 2 tests  27b: 2 tests  30: 3 tests  33: 5 tests  34: 2 tests  50: 4 tests | BB00 vs YY00  YY00 vs BY00  BY00 vs YY00  BY00 vs YY00  B0B0 vs Y0B0  B00B vs Y00B  0B0Y vs 0Y0Y | 2  2  2  3  2  2  3 | BB00: 23/4  YY00: 7/21  YY00: 21/5  BY00: 5/20  BY00: 10/4  YY00: 2/13  BY00: 19/4  YY00: 9/30  B0B0: 28/2  Y0B0: 4/25  B00B: 12/1  Y00B: 3/14  0B0Y: 32/10  0Y0Y: 6/25 | 85%  75%  81%  80%  71%  87%  83%  77%  93%  86%  92%  82%  76%  80% |
| 10 | 19a: 2 test  20: 3 tests  23: 2 tests  24: 4 tests  31: 5 tests  35: 1 test  51: 4 tests | B0Y0 vs B0B0  00BB vs 00BY  BB00 vs BY00  YY00 vs YB00  0BB0 vs 0BY0  B00B vs B00Y  0B0B vs 0B0Y | 2  3  2  3  3  2  3 | B0Y0: 11/10  B0B0: 12/11  00BB: 14/17  00BY: 15/17  BB00:18/7  BY00: 8/19  YY00:16/4  YB00: 8/18  0BB0: 33/19  0BY0: 19/31  B00B: 4/5  B00Y: 6/8  0B0B: 18/20  0B0Y: 18/19 | 52%  48%  45%  53%  72%  70%  80%  72%  64%  62%  44%  57%  47%  51% |
| 11 | 19b: 2 tests  21: 4 tests  22: 2 tests  27a: 2 tests  29: 4 tests  42: 4 tests  52: 4 tests | B0B0 vs Y0Y0  YB00 vs BY00  0YB0 vs 0BY0  BB00 vs YY00  00YB vs 00BY  0B0Y vs 0Y0B  Y00B vs B00Y | 3  3  2  2  3  3  3 | B0B0: 13/5  Y0Y0: 4/12  YB00:29/8  BY00: 4/20  0YB0: 13/6  0BY0: 5/16  BB00: 16/2  YY00: 6/22  00YB: 15/14  00BY:16/20  0B0Y: 25/10  0Y0B: 8/20  Y00B: 24/11  B00Y: 10/24 | 72%  71%  78%  83%  68%  76%  88%  79%  52%  56%  71%  71%  69%  71% |
| 12 | 36: 3 tests  38: 3 tests | BYB0 vs YBY0  YYB0 vs BBY0 | 2  3 | BYB0: 21/2  YBY0: 1/17  YYB0: 27/5  BBY: 6/22 | 91%  94%  84%  79% |
| 13 | 9: 3 tests  13: 3 tests  40: 4 tests | BYB0 vs YBB0  BYB0 vs Ybb0  BBY0 vs YYY0 | 3  3  3 | BYB0: 17/6  YBB0: 5/15  BYB0: 18/6  YYY0:1/18  BBY0: 22/3  YYY0: 4/28 | 74%  75%  75%  95%  88%  88% |
| 14 | 11: 3 tests  36a: 4 tests | BYB0 vs YYY0  BBY0 vs YBB0 | 4  4 | BYB0: 29/7  YYY0: 3/21  BBY0: 27/13  YBB0: 10/22 | 81%  88%  68%  69% |
| 15 | 10: 3 tests  41: 4 tests | BYB0 vs YBB0  BYB0 vs YYB0 | 4  4 | BYB0: 17/6  YBB0: 5/15  BYB0: 25/11  YYB0: 3/19 | 74%  75%  69%  86% |
| 16 | 14: tests  33a: 3 tests | BYB0 vs BBY0  YYB0 vs YBY0 | 3  3 | BYB0: 28/14  BBY0: 15/18  YYB0: 19/10  YBY0: 7/11 | 67%  56%  66%  61% |
| 17 | 37: 6 tests  42a: 3 tests | BBY0 vs BYY0  YYB0 vs YBB0 | 3  3 | BBY0:23/18  BYY0: 10/26  YYB0: 14/7  YBB0: 7/16 | 56%  72%  67%  70% |
| 18 | 12: 4 tests  39: 4 tests | BYB0 vs BYY0  YYB0 vs YYY0 | 2  3 | BYB0: 12/13  BYY0: 11/5  YYB0: 16/19  YYY0: 22/22 | 48%  69%  46%  50% |
