# Supplementary material for: Serial Position Learning in Honeybees
Source: PLoS One. 2009 Mar 4;4(3):e4694. doi: 10.1371/journal.pone.0004694 (PMC2649506; doi:10.1371/journal.pone.0004694)
Supplement: Table S2 — Transfer tests. Rows and columns a, b, c and d are the same as in table S1. Column e gives the patterns presented during the transfer tests together with the choices for these patterns. As in table S1 the choices of the left arm are shown first and then the ones for the right arm of the T-maze. Column f gives the % of choices for the left arm. (0.05 MB DOC) [file pone.0004694.s002.doc]

Table S2: transfer tests

| a | b | c | d | e | f |
| --- | --- | --- | --- | --- | --- |
| column in  Table 1 B | number of experiment:  number of tests | training patterns  (left vs right site) | number  of animals | sum of choices in all transfer tests (left/right) | % choices left |
| 1 | 2: 2 tests | 0B00 vs BBBB | 2 | 0Y00: 13/0  YYYY: 3/16 | 100%  16% |
| 2 | 6: 1 test | 0Y00 vs YYYY | 2 | 0B00: 7/2  BBBB: 2/8 | 78%  11% |
| 3 | 21: 3 tests | YB00 vs BY00 | 3 | BB00: 1/24  YY00: 29/2 | 4%  94% |
| 4 | 9: 2 | BYB0 vs YBB0 | 3 | 0BYB: 9/5  0YBB: 5/15 | 64%  25% |
| 5 | 6: 2 tests | 0Y00 vs YYYY | 2 | 0YY0: 18/3  000Y: 8/2 | 86%  80% |
| 6 | 15:2 tests each | B000 vs Y000 | 2 | 0B00: 6/0  0Y00: 0/6  000B: 12/7  000Y: 6/9 | 100%  0%  63%  60% |
| 7 | 22: 1 test each | 0YB0 vs 0BY0 | 2 | 00YB: 4/5  00BY: 7/6  YB00: 6/1  BY00: 2/5 | 44%  54%  86%  25% |
| 8 | 20: 2 tests each | BB00 vs YB00 | 3 | BB00: 11/12  BY00: 13/10 | 48%  57% |
| 9 | 26: 2 tests each | YY00 vs BY00 | 2 | 0BY0: 9/18  00BY: 3/15  0YY0: 15/2  00YY: 13/2 | 33%  17%  88%  87% |
| 10 | 30: 2 tests each | 0BY0 vs 0YY0 | 3 | BY00: 22/16  00BY: 13/8  YY00: 21/26  00YY: 5/15 | 58%  62%  55%  25% |
| 11 | 31: 2 tests each | 0BB0 vs 0BY0 | 3 | BY00: 17/18  BB00: 15/26  00BY: 9/8  00BB: 8/9 | 49%  37%  53%  47% |
| 12 | 33: 2 tests each | B0B0 vs Y0B0 | 2 | 0B0B: 14/9  0Y0B: 19/15 | 61%  40% |
| 13 | 34: 1 test each | B00B vs Y00B | 2 | 0B0B: 5/4  0Y0B: 3/5 | 56%  63% |
| 14 | 9: 2 tests each | BYB0 vs YBB0 | 3 | 0BYB: 9/5  0YBB: 5/15 | 64%  25% |
| 15 | 36: 2 tests each | BYB0 vs YBY0 | 2 | 0BYB: 16/7  0YBY: 7/15 | 70%  25% |
| 16 | 2: 3 tests each | 0B00 vs BBBB | 2 | 00BB: 21/3  BB00: 6/21  B00B: 1/27  0B00: 13/2  00B0: 5/2  000B: 13/0  B000: 5/13  0BB0: 15/0  0YY0: 6/0  Y000: 10/22 | 88%  22%  4%  87%  71%  100%  28%  100%  100%  31% |
